# Mumps Virus Induces Protein-Kinase-R-Dependent Stress Granules, Partly Suppressing Type III Interferon Production

**DOI:** 10.1371/journal.pone.0161793

**Published:** 2016-08-25

**Authors:** Shin Hashimoto, Soh Yamamoto, Noriko Ogasawara, Toyotaka Sato, Keisuke Yamamoto, Hiroshi Katoh, Toru Kubota, Tsukasa Shiraishi, Takashi Kojima, Tetsuo Himi, Hiroyuki Tsutsumi, Shin-ichi Yokota

**Affiliations:** 1 Department of Pediatrics, Sapporo Medical University School of Medicine, Sapporo, Japan; 2 Department of Microbiology, Sapporo Medical University School of Medicine, Sapporo, Japan; 3 Department of Otorhinolaryngology, Sapporo Medical University School of Medicine, Sapporo, Japan; 4 Department of Virology III, National Institute of Infectious Diseases, Tokyo, Japan; 5 Department of Cell Science, Research Institute for Frontier Medicine, Sapporo Medical University School of Medicine, Sapporo, Japan; University of Missouri Columbia, UNITED STATES

## Abstract

Stress granules (SGs) are cytoplasmic granular aggregations that are induced by cellular stress, including viral infection. SGs have opposing antiviral and proviral roles, which depend on virus species. The exact function of SGs during viral infection is not fully understood. Here, we showed that mumps virus (MuV) induced SGs depending on activation of protein kinase R (PKR). MuV infection strongly induced interferon (IFN)-λ1, 2 and 3, and IFN-β through activation of IFN regulatory factor 3 (IRF3) via retinoic acid inducible gene-I (RIG-I) and the mitochondrial antiviral signaling (MAVS) pathway. MuV-induced IFNs were strongly upregulated in PKR-knockdown cells. MuV-induced SG formation was suppressed by knockdown of PKR and SG marker proteins, Ras-GTPase-activating protein SH3-domain-binding protein 1 and T-cell-restricted intracellular antigen-1, and significantly increased the levels of MuV-induced IFN-λ1. However, viral titer was not altered by suppression of SG formation. PKR was required for induction of SGs by MuV infection and regulated type III IFN (IFN-λ1) mRNA stability. MuV-induced SGs partly suppressed type III IFN production by MuV; however, the limited suppression was not sufficient to inhibit MuV replication in cell culture. Our results provide insight into the relationship between SGs and IFN production induced by MuV infection.

## Introduction

Mumps is an infectious disease caused by mumps virus (MuV) and is characterized by swelling of the parotid gland [1]. Mumps has severe characteristic complications such as aseptic meningitis, encephalitis, severe sensory hearing loss, pancreatitis and orchitis. The disease can be prevented by vaccination with attenuated live vaccine, which is used universally in many countries around the world. MuV is an enveloped single negative strand RNA virus that belongs to the genus Rubulavirus in the family Paramyxoviridae [1, 2]. MuV particles consist of seven proteins, N, P, M, F, SH, HN and L [3, 4]. V protein, which is encoded by P gene, is a nonstructural protein, and it strongly inhibits interferon (IFN) signal transduction, resulting in shutoff of the IFN-induced host antiviral response [5].

The innate immune response is known to be one of the most important defense mechanisms against pathogenic bacteria, viruses, and foreign antigens. The innate immune sensors in host cells, called pattern recognition receptors (PRRs), detect pathogen-associated molecular patterns and initiate antimicrobial immune responses [6]. PRRs contain several well-defined systems: Toll-like receptors; retinoic acid inducible gene-I (RIG-I)-like receptors (RLRs); and cytoplasmic DNA sensors such as DNA-dependent activator of IFN-regulatory factors. Viral RNAs are mainly recognized by RLRs, and signals are transmitted to the mitochondrial antiviral signaling (MAVS) pathway, which is localized on the mitochondrial outer membrane [7]. RLR/MAVS interaction activates the IFN regulatory factors (IRFs) via activation of the TANK-binding kinase 1/inducible IκB kinase (IKK) pathways and nuclear factor (NF)-κB via activation of the IKKα/IKKβ pathway. Activated IRFs and NF-κB induce transcription of IFNs and proinflammatory cytokines [8]. IFNs induce expression of antiviral factors called IFN-stimulated genes (ISGs), such as myxovirus resistance A (MxA) and 2′-5′-oligoadenylate synthetase, through the Janus kinase (JAK)–signal transducer and activator of transcription (STAT) pathway, and prevent viral replication [9].

Cellular stress, such as heat shock, hypoxia, and viral infection, induces formation of cytoplasmic granules called stress granules (SGs) [10]. SGs are ribonucleoprotein aggregates that contain stalled 48S initiation complexes and various RNA-binding proteins, such as Ras-GTPase-activating protein SH3-domain-binding protein (G3BP)1, T-cell-restricted intracellular antigen (TIA)-1, and TIA-1-related protein (TIAR) [11]. SGs are temporary storage sites for translationally stalled mRNAs, and are associated with regulation of host mRNA translation. Typically, formation of SGs is initiated from phosphorylation of eukaryotic translation initiation factor 2α (eIF2α). There are four well-known kinases that phosphorylate eIF2α: double-stranded (ds)RNA-dependent protein kinase (PKR) [12]; PKR-like endoplasmic reticulum kinase (PERK) [13]; general control non-derepressible 2 (GCN2) [14]; and heme-regulated eIF2α kinase (HRI) [15]. Some viruses induce SGs, which influence IFN production and viral replication [10, 16, 17]. In contrast, some viruses, such as influenza A virus (IAV), measles virus (MeV) and Sendai virus (SeV), block SG formation and inhibit IFN production [18–20]. This suggests that SG formation is one of the defense mechanisms against viral invasion in host cells. However, the specific role or function of SGs is not yet well defined. In addition, it has not been reported whether SGs are induced by MuV infection.

In the present study, we demonstrated that MuV-induced SG formation was dependent on PKR. The PKR-dependent SGs partly suppressed production of IFN, especially IFN-λ; however, this did not affect viral replication. We discuss the relationship between SG formation and MuV-induced IFNs.

## Materials and Methods

### Antibodies and Reagents

Rabbit monoclonal antibodies (mAbs) against phospho-(P-)IRF3(Ser96) (4D4G), eIF2α(D7D3) and P-eIF2α(Ser51) (D9D8), and rabbit polyclonal antibody (PcAb) against PKR (3072) were purchased from Cell Signaling Technology (Danvers, MA). Rabbit mAb against P-PKR(Thr451) (EPR2152Y) (ab81303) and mouse mAb against G3BP1 (ab56574) were purchased from Abcam (Cambridge, UK). Rabbit PcAbs against IRF3 (309033), RIG-I (PAB12973), melanoma differentiation-associated gene 5 (MDA5) (29020), Hu-antigen R (HuR), and insulin-like growth factor 2 binding protein (IGF2BP)1 were purchased from Active Motif (Carlsbad, CA), Abnova (Taipei, Taiwan), Immuno-Biological Laboratories (Gunma, Japan), Medical and Biological Laboratory (Nagoya, Japan) and Abgent (San Diego, CA), respectively. Mouse mAbs against MAVS (E-3) and β-actin (AC-74) were purchased from Santa Cruz Biotechnology (Dallas, TX) and Sigma–Aldrich (St. Louis, MO), respectively. Rabbit PcAb against mumps P/V protein (T61), and mouse mAb against mumps F protein (44C) were generated as previously described [21–23]. Actinomycin D was purchased from Wako Pure Chemicals (Osaka, Japan) and dissolved with dimethyl sulfoxide.

### Cells and Viruses

FL (human amnion) and Vero (African green monkey kidney) cells were obtained from American Type Culture Collection (ATCC; Manassas, VA), and were maintained in RPMI-1640 medium supplemented with 10% (v/v) fetal bovine serum, 100 U/ml penicillin and 10 μg/ml streptomycin. BEAS-2 (human bronchial epithelial) cells were obtained from ATCC, and maintained in LHC-9 medium supplemented with 100 U/ml penicillin and 10 μg/ml streptomycin. The MuV strain Torii was grown in Vero cells, and supernatant containing MuV was frozen in liquid nitrogen and stored at −80°C until use. Cells were infected with MuV at a multiplicity of infection (MOI) of 0.005–0.5 at 37°C for 1 h. After infection, cells were washed twice with prewarmed medium and then incubated for 24 h. The viral titers in supernatants were determined by plaque-forming assays in Vero cells as follows. Samples serially diluted 10-fold were adsorbed onto Vero cells in 24-well plates at 37°C for 1 h. After incubation, infected Vero cells were washed twice with phosphate-buffered saline (PBS) and incubated in culture medium supplemented with 1% carboxymethyl cellulose for 6 days. After incubation, cells were stained with staining solution [0.5% (w/v) crystal violet, 5% (v/v) formalin, 50% (v/v) ethanol, and 0.15 M NaCl]. Experimental methods and preparation of human respiratory syncytial virus (RSV) were as described previously [24].

### Immunocytochemistry

The cells infected with MuV were grown in a 35-mm glass-bottom dish coated with type I collagen (Matsunami Glass, Kishiwada, Japan), and fixed with 4% paraformaldehyde at room temperature for 15 min. After rinsing in PBS, the cells were treated with PBS containing 0.1% Triton X-100 for 5 min, and then with blocking buffer (PBS containing 2% bovine serum albumin and 0.02% Triton X-100) for 20 min at room temperature. Cells were incubated with primary antibodies for 2 h at room temperature. After rinsing in PBS, the specimens were incubated with secondary antibodies, Alexa Fluor 488 (green)-conjugated anti-rabbit IgG or Alexa Fluor 594 (red)-conjugated anti-mouse IgG (Thermo Fisher Scientific, Waltham, MA), for 2 h at room temperature. Nuclei were stained with 4′,6-diamino-2-phenylindole (DAPI) for 2 h at room temperature. After the stained slides were washed with PBS, the cells were embedded in 2% 1,4-diazabicycle [2.2.2.] octane (Sigma–Aldrich) and 50% glycerol in PBS. The specimens were examined and photographed using confocal microscopy (ConfoCor 3 LSM 510 META; Carl Zeiss, Oberkochen, Germany).

### Measurement of Cytokine Production

Cytokines in cell culture supernatants were determined using an enzyme-linked immunosorbent assay (ELISA). Human IFN-λ1, 2 and 3 were measured using respective DuoSet ELISA Development Systems (R&D Systems, Minneapolis, MN). Human IFN-β ELISA kit was purchased from Kamakura Techno-Science (Kamakura, Japan).

### RNA Interference

Silencer selected small-interfering RNAs (siRNAs) were obtained from Thermo Fisher Scientific. Targeting siRNA was transfected using Lipofectamine RNAimax Transfection Reagent (Thermo Fisher Scientific).

### RNA Analysis

Total RNA was purified using an RNeasy Plus Mini Kit (Qiagen, Hilden, Germany). Reverse transcription polymerase chain reactions (RT-PCRs) were performed using the Onestep RT-PCR kit (Qiagen) and specific primers as below: MuV F forward, 5′-cggtctaatggagggtcaga-3′ and reverse 5′-cagtgctacaaatcgcctca-3′; MuV P/V forward, 5′-cattcagggaaccaactcgt-3′ and reverse 5′-aaattctgttaccggcgttg-3′. cDNA was synthesized using SuperScript III First-Strand Synthesis Kit (Thermo Fisher Scientific). Primer sets of IFN-λ1 and Mx1 were purchased from Thermo Fisher Scientific. Hypoxanthine phosphoribosyltransferase (HPRT)1 and IFN-β primer sets for 5′ nuclease assay were synthesized by Integrated DNA Technologies (Coralville, IA). Primer set and probe sequence of IFN-β were: 5′-/56-FAM/CAGACAAGA/ZEN/TTCATCTAGCACTGGCTGG/3lABkFQ/-3′, forward, 5′-GCCGCATTGACCATCTATG-3′, and reverse, 5′-GCCAGGAGGTTCTCAACAATAG-3′. G3BP1 and TIA-1 mRNA was quantified using specific primers: G3BP1 forward 5′-GCAGATGCAGTCTACGGACA-3′, reverse 5′-TCACCTGGACTACCACACCA-3′ and TIA-1 forward 5′-AAGCTGCTTTTGCACCATTT-3′ and reverse 5′-TTCAGCATCCCATTTGTTGA-3′. Real-time PCR was performed using Thunderbird Probe qPCR Mix or KOD SYBR qPCR mix (Toyobo, Osaka, Japan) by LightCycler480 (Roche, Basel, Switzerland). mRNA was normalized with HPRT1 and relative quantitation was calculated by ΔΔCt methods.

### Reporter Gene Assay

Reporter plasmids pIFN-βP125-Luc and pPRDIII-I-Luc were constructed as described previously [24]. pGL3-ELAM-L, which contains the promoter region of E-selectin [endothelial-leukocyte adhesion molecule (ELAM)1], was kindly provided by Dr. Masashi Muroi (Musashino University, Nishitokyo, Japan). Reporter plasmid (90 ng/well) was transfected with pRL-TK (10 ng/well) (Promega, Madison, WI), which was used as a reference, using Lipofectamine 3000 Reagent (Thermo Fisher Scientific). Luciferase activity 24 h post-infection was measured using the Dual-Luciferase Reporter Assay System (Promega) and Infinite M1000 Pro (Tecan, Mannedorf, Switzerland).

### Western Blotting

Cells were lysed with lysis buffer (1% Triton X-100, 0.5% sodium deoxycholate, 0.15 M NaCl, and 2 mM EDTA in 50 mM HEPES–NaOH, pH 7.4) supplemented with protease inhibitor cocktail (Thermo Fisher Scientific). After centrifugation at 20,000 × *g* for 5 min, protein concentration in supernatant was determined using the bicinchoninic acid method (Thermo Fisher Scientific). Sodium dodecyl sulfate polyacrylamide gel electrophoresis and western blotting were performed as described previously [25].

### Statistical Analysis

Digital images of western blotting were quantitated using public domain Image J program (National Institutes of Health, Bethesda, MD). The significance of differences between two groups was analyzed by two-tailed Student’s *t* test.

## Results

### MuV-Induced SG Formation

**Fig 1:**
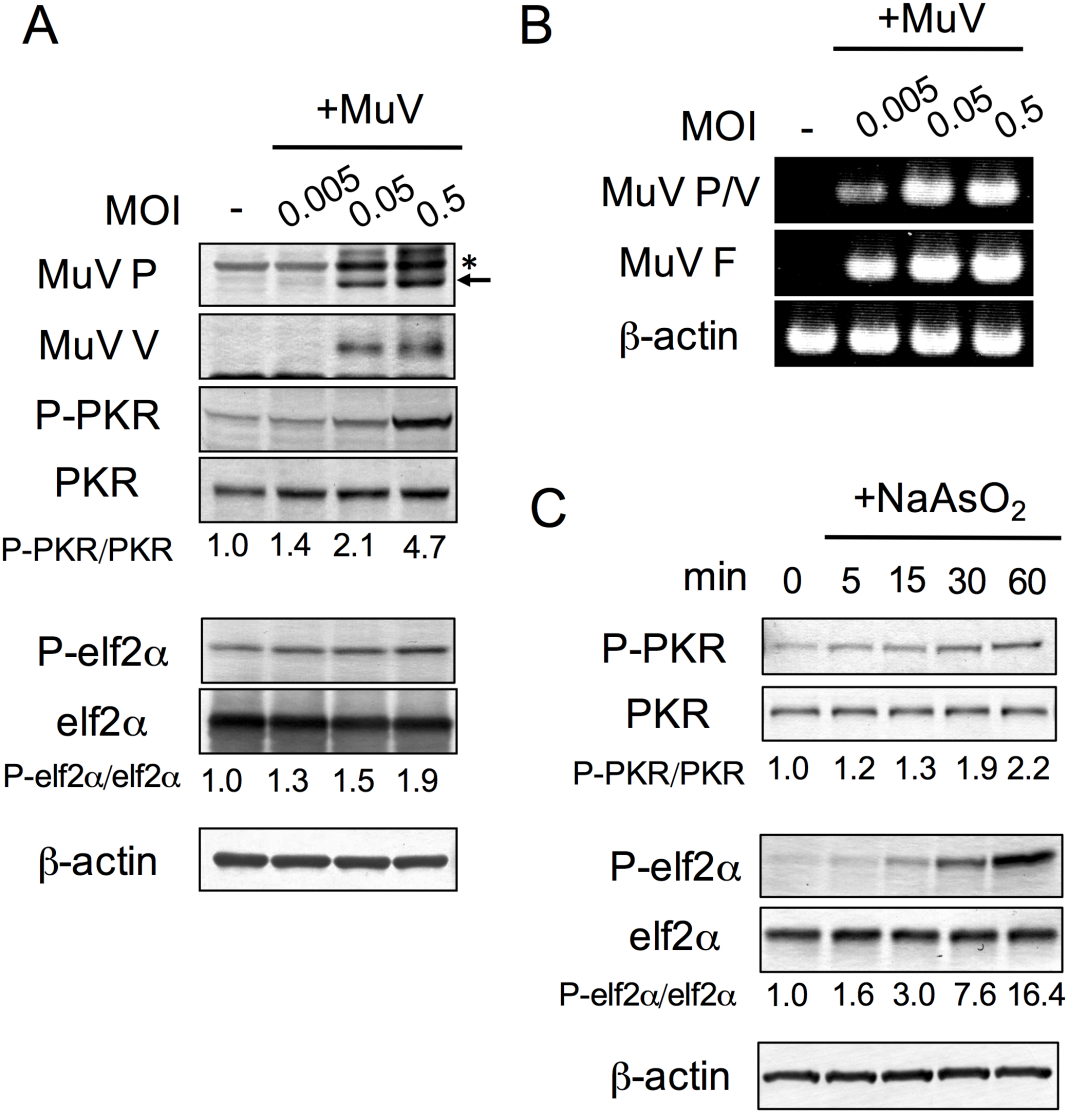
Induction of PKR and eIF2α phosphorylation by MuV infection and treatment with sodium arsenite. FL cells were infected with MuV at various MOI. The cells were analyzed at 24 h post-infection. β-Actin was determined as a control. (A) Western blotting of viral and host proteins. Phosphorylation ratio was expressed as the value of uninfected FL cells, which was set to 1. Arrow indicates the band of MuV P protein, and asterisk indicates a nonspecific band. (B) RT-PCR analysis of viral mRNAs. (C) Western blotting of FL cells treated with 0.14 mM sodium arsenite. Phosphorylation ratio was expressed as the value of untreated FL cells, which was set to 1.

We examined whether MuV induced SGs. In MuV-infected cells, at 24 h post-infection, MuV P and V proteins were detectable at MOI >0.05, and mRNAs of P/V and F at MOI >0.005 (Fig 1A and 1B). PKR and eIF2α are regulators of SGs. PKR was strongly phosphorylated by MuV infection, whereas eIF2α was only weakly phosphorylated (Fig 1A). As a reference, we used sodium arsenite, which induces oxidative stress and activation of HRI [15, 26]. Sodium arsenite induced phosphorylation of eIF2α after 15 min treatment, which was dramatically increased after 1 h treatment (Fig 1C). Phosphorylation of eIF2α induced by MuV, which was mainly mediated by PKR, was less than that induced by sodium arsenite. These results suggested that MuV infection led to moderate shutoff of protein translation, whereas sodium arsenite showed the strong potency of shutoff of protein translation in FL cells (Fig 1C).

**Fig 2:**
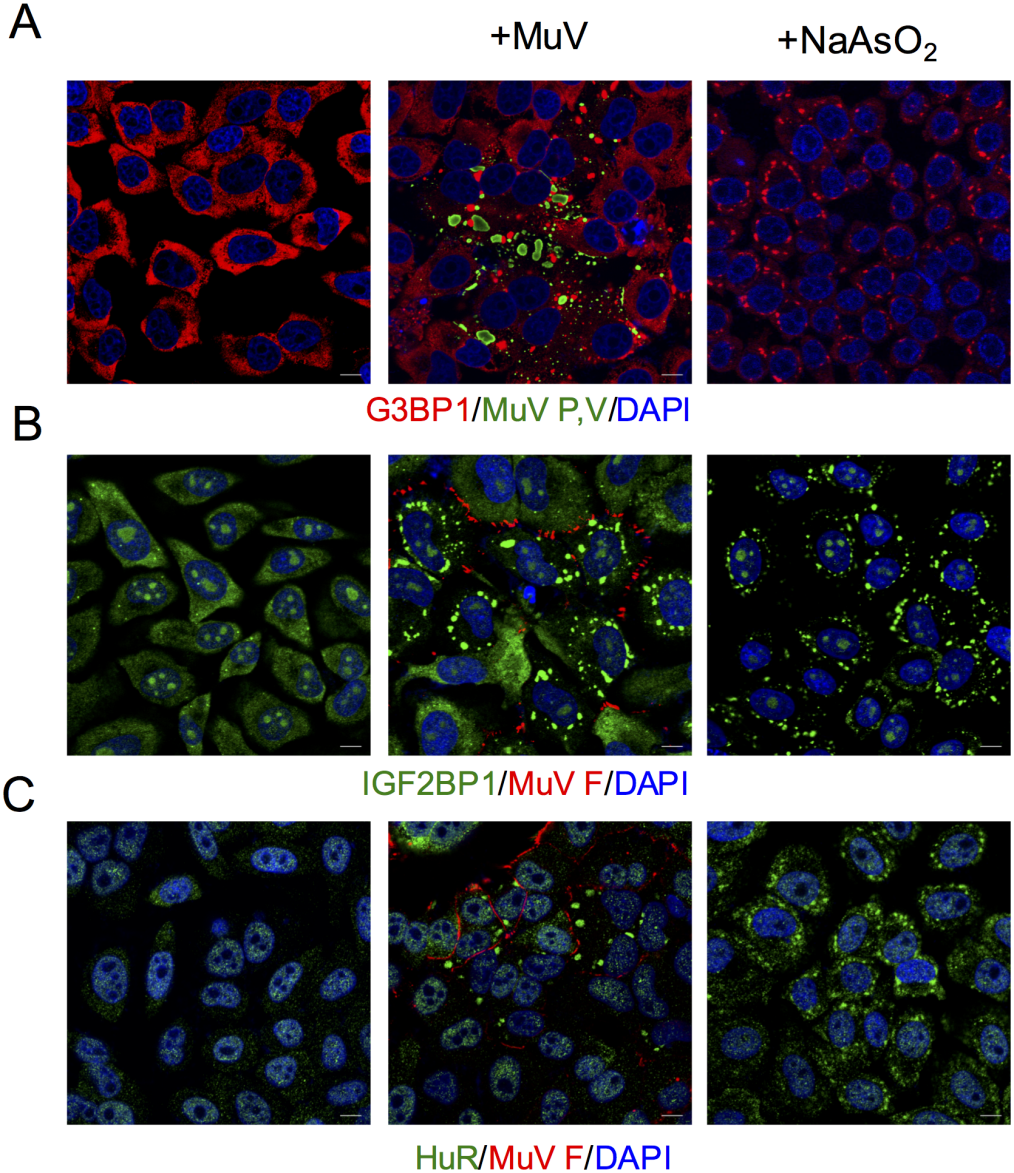
MuV-induced SGs. Immunofluorescence staining of (A) G3BP1 (red) and MuV P/V proteins (green), (B) IGF2BP1 (green) and MuV F proteins (red), and (C) HuR (green) and MuV F proteins (red). FL cells were infected with MuV at MOI 0.5. Cells were analyzed by immunofluorescence microscopy at 24 h post-infection. Left panels: untreated cells. Center panels: MuV-infected cells. Right panels: cells treated with 0.14 mM sodium arsenite for 30 min as a control for SG formation. Nuclei were stained with DAPI (blue). Scale bar indicate 10 μm.

We confirmed that foci (aggregations) containing SG marker proteins, namely G3BP1, IGF2BP1 and HuR (also known as ELAV-like protein) [10, 27], were induced by sodium arsenite (Fig 2). Similar foci were observed in the cytosol of MuV-infected cells, while G3BP1 and IGF2BP1 were mainly localized in the cytosol and HuR in the nucleus in untreated cells (Fig 2). MuV P/V proteins were observed as inclusion bodies in the cytosol, and MuV F protein was in the cell membrane. MuV P/V and F proteins did not colocalize with the SG markers. We also observed that MuV-induced foci contained G3BP1 and upregulation of phosphorylation of PKR in human bronchial epithelium cell line BEAS-2B (data not shown). These results indicated that MuV induced formation of SGs.

### Relationship between SG Formation and IFN Response

**Fig 3:**
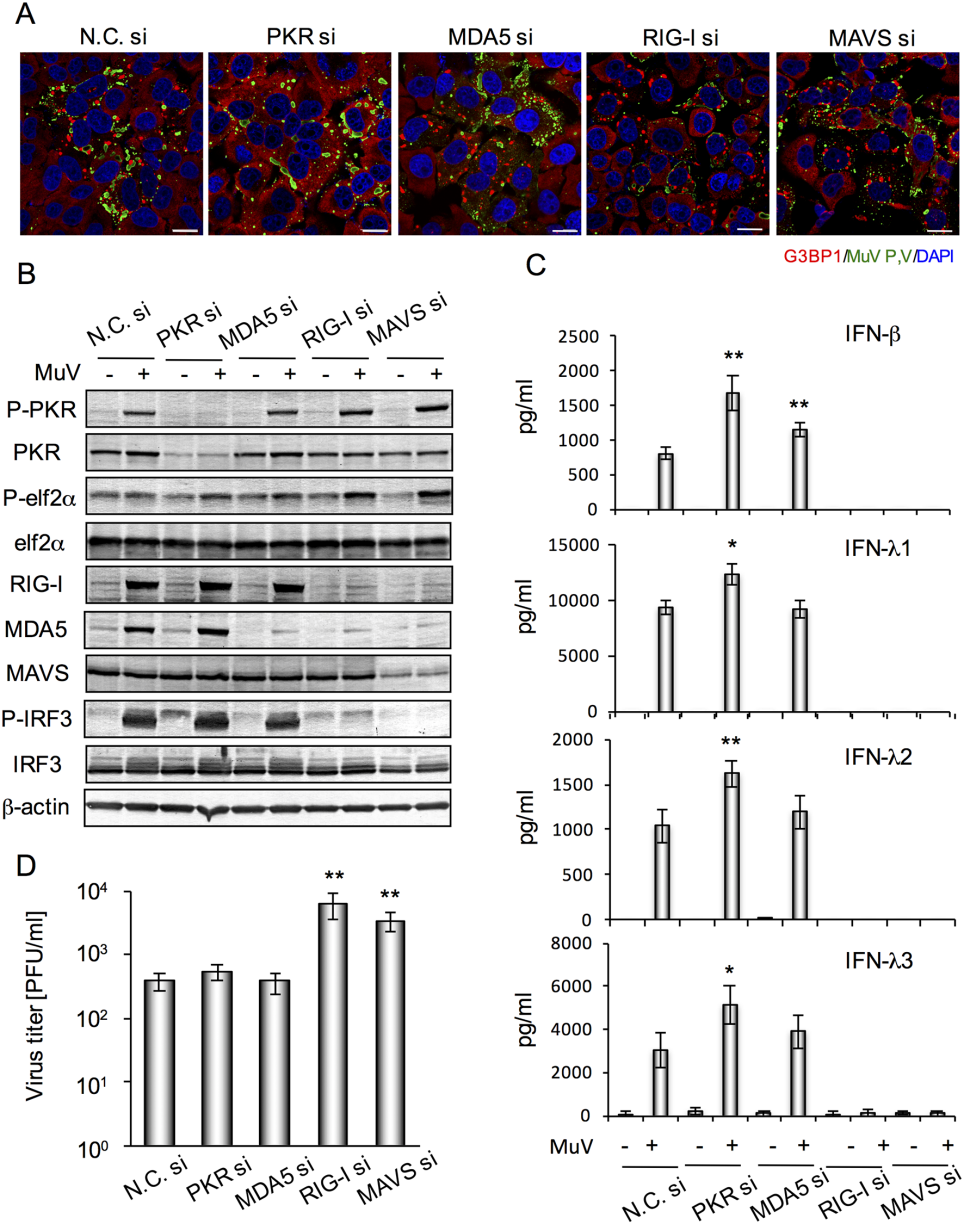
Relationship between MuV-induced SG formation, IFN production and viral replication in PKR-KD and RLR-related protein-KD cells. FL cells were transfected with targeting siRNA (PKR si, MDA5 si, RIG-I si, or MAVS si) or nontargeting control siRNA (N.C. si) for 48 h, and then infected with MuV at MOI 0.5. (A) Immunofluorescence staining of G3BP1 (red) and MuV P/V proteins (green) at 24 h post-infection of MuV. Nuclei were stained with DAPI (blue). Scale bar indicates 20 μm. (B) Western blotting of whole cell lysates derived from cells at 24 h post-infection. β-Actin was determined as a control. (C) IFNs (*n* = 4) determined by ELISA and (D) viral titer (*n* = 4) determined by plaque-forming assay in culture supernatants at 24 h post-infection at MOI 0.5. The bar graphs represent means ± standard deviation. *P<0.05, **P<0.01 versus MuV-infected control cells (N.C. siRNA-transfected cells).

Virus-induced SGs are reported to affect IFN induction and viral replication [10]. We investigated the contribution of MuV-induced SGs to viral replication and IFN production, and their relationship with RLR-related proteins, such as RIG-I, MDA5 and MAVS. We examined SG formation in PKR-, RIG-I-, MDA5- and MAVS-knockdown (KD) cells. In all KD cells, SGs were formed by treatment with sodium arsenite (data not shown). MuV-induced SGs were not observed in PKR-KD cells, while they were observed in RIG-I-, MDA5- and MAVS-KD cells, similar to control cells (Fig 3A). SGs containing G3BP1 were observed in ~80% of MuV-infected control cells. Phosphorylation of PKR was stimulated by MuV infection in the MDA5-, RIG-I-, MAVS-KD as well as control cells (Fig 3B). eIF2α was phosphorylated by MuV infection in all KD cells. eIF2α is a substrate protein of PKR; however, it was phosphorylated by MuV infection in PKR-KD cells. The results suggested that MuV induced eIF2α phosphorylation in a PKR-independent manner.

We determined the production of IFNs in the KD cells (Fig 3C). IFN production by MuV almost completely disappeared in RIG-I- and MAVS-KD cells. It was not altered in MDA5-KD cells. MuV-infection-induced production of IFN-β, -λ1, -λ2 and -λ3 was significantly higher in PKR-KD cells, as compared with the control cells (Fig 3C). IRF3, which is the main transcription factor for production of IFN-β and IFN-λs, was clearly phosphorylated by MuV infection, but not in MAVS- and RIG-I-KD cells. MDA5 and RIG-I induced by MuV infection disappeared in MAVS-KD cells (Fig 3B). Virus titer in the culture supernatants of RIG-I- and MAVS-KD cells significantly increased in comparison to that in control cells, while viral titer was not altered in PKR- and MDA5-KD cells (Fig 3D). These results indicated that MuV was recognized by RIG-I but not MDA5, and produced type I and III IFNs through a MAVS- and IRF3-dependent signal transduction pathway that suppressed viral replication. SG formation induced by MuV was dependent on PKR-dependent, but not RLR-dependent signal transduction. There was evidence that PKR-dependent SG formation partly suppressed IFN production in MuV-infected cells.

**Fig 4:**
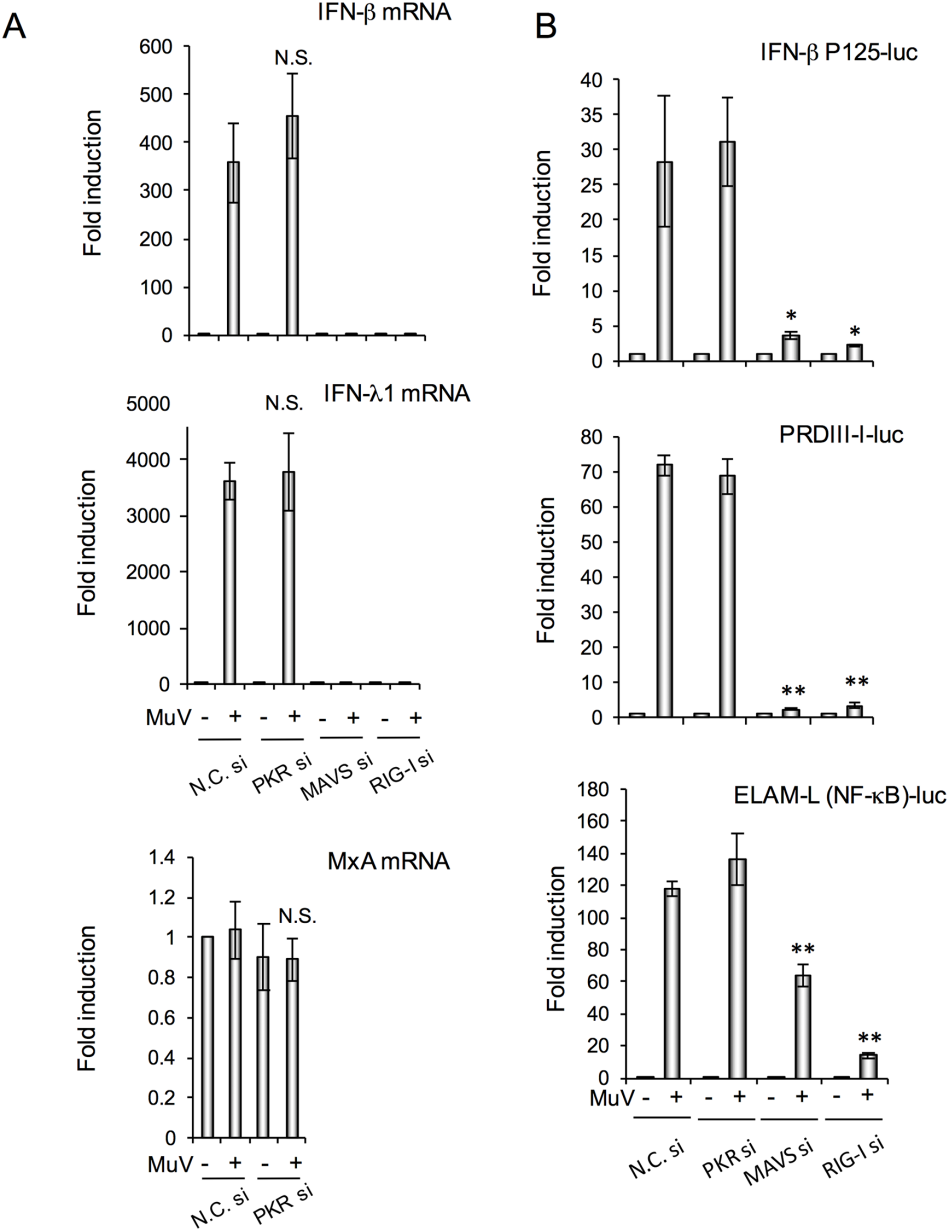
MAVS and RIG-I, but not PKR, regulated IFN induction at the transcription level. FL cells were transfected with targeting siRNA (PKR si, MAVS si, or RIG-I si) or nontargeting control siRNA (N.C. si) for 48 h, and then infected with MuV at MOI 0.5. (A) MuV-induced mRNAs (IFN-β, IFN-λ1 and MxA) at 12 h post-infection determined by real-time RT-PCR (*n* = 4). (B) Luciferase activity of reporter gene assay (*n* = 3) for IFN-β promoter (IFN-β P125), IRF binding element (PRDIII-I), and NF-κB binding element (ELAM) at 24 h post-infection. The bar graphs represent means ± standard deviation. *P<0.05, **P<0.01 versus MuV-infected control cells (N.C. siRNA-transfected cells).

We investigated induction of IFN mRNA and activation of transcription factors in the KD cells (Fig 4). mRNA of IFN-β and -λ1 was markedly induced at 12 h post-infection, but not in MAVS- and RIG-I-KD cells (Fig 4A). The ISG MxA was not stimulated by MuV infection. IFN and MxA mRNA expression in PKR-KD cells was not altered in comparison to control cells (Fig 4A). The activity of IRF and NF-κB was measured by reporter gene assay using a luciferase expression plasmid containing IFN-β p125 promoter, PRD III-I (an IRF-binding sequence), and ELAM promoter (mainly regulated by NF-κB [28]) (Fig 4B). Luciferase activity was stimulated in control and PKR-KD cells by MuV infection at comparable levels. In MAVS- and RIG-I-KD cells, MuV-stimulated IFN-β promoter and PRD III-I activities were almost completely suppressed (Fig 4B). MuV-stimulated NF-κB activity was markedly decreased in RIG-KD cells, and decreased ~50% in MAVS-KD cells compared with control cells. These data suggest that PKR is not directly related to NF-κB and IRF3 activation via the RIG-I/MAVS/IRF3 signaling cascade in MuV-infected cells.

### PKR Regulated IFN-λ1 mRNA Stability

**Fig 5:**
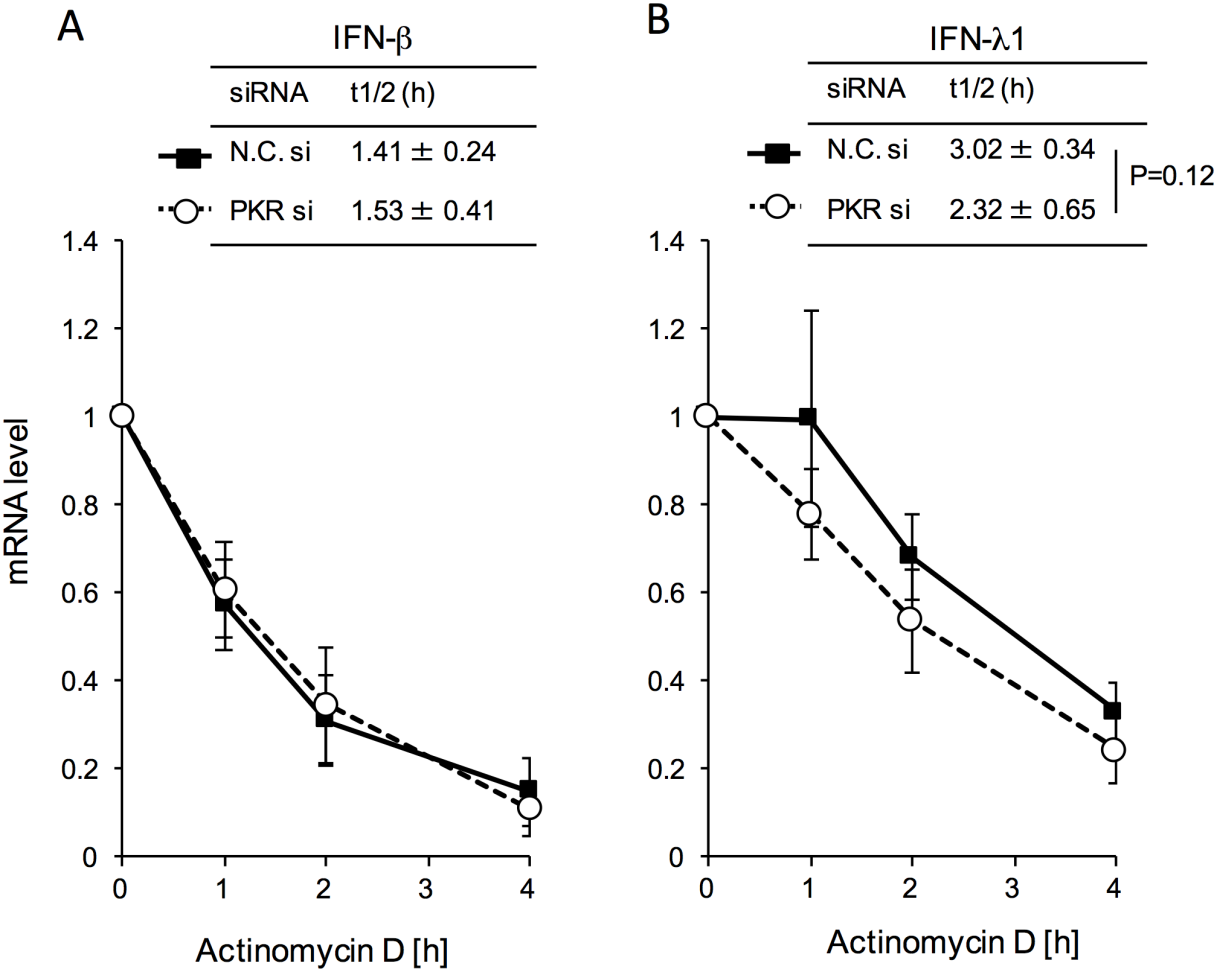
PKR regulated mRNA stability of IFN-λ1, but not IFN-β. FL cells were transfected with targeting siRNA for PKR (PKR si) or nontargeting control siRNA (N.C. si) for 48 h, and then infected with MuV at MOI 0.5. Twenty hours post-infection, actinomycin D (5 μM) was added. (A) IFN-βand (B) IFN-**λ**1 mRNA levels at each time were determined by real-time RT-PCR (*n* = 4). Amount of IFN mRNA in cells without actinomycin D treatment was set to 1. Empty circle/dotted line and filled square/solid line indicate PKR-targeting siRNA transfected cells and nontargeting control siRNA transfected cells, respectively.

Compared with control cells, increased MuV-induced IFN protein production was observed only in PKR-KD cells (Fig 3C). The levels of MuV-induced IFN-β and -λ1 mRNAs were not significantly altered by PKR-KD (Fig 4A). IFN-β mRNA was induced by 337.8- and 430.1-fold in control and PKR-KD cells, respectively, and IFN-λ1 mRNA was induced by 3119- and 3384-fold in control and PKR-KD cells, respectively (Fig 4A). SGs are known to regulate mRNA localization, stability and translation after transcription [29]. We therefore hypothesized that PKR controls the stability of IFN mRNA induced by MuV infection through SGs. The half-lives of mRNAs were determined by real-time RT-PCR after terminating transcription by actinomycin D, a transcription inhibitor, which does not alter and induce SGs [30] (Fig 5). IFN mRNAs in uninfected cells treated with actinomycin D were undetectable, so we could not determine their half-lives. We calculated half-lives of IFN-β and -λ1 mRNAs at 24 h post-infection. Half-life of IFN-λ1 mRNA was 1.5 h longer than that of IFN-β mRNA (*P*<0.001) in control cells infected with MuV. Half-lives of IFN-β mRNA in control and PKR-KD cells infected with MuV were similar: 1.41 ± 0.24 and 1.53 ± 0.41 h, respectively. Half-life of IFN-λ1 mRNA in MuV-infected PKR-KD cells was shorter than that of MuV-infected control cells: 2.32 ± 0.65 h and 3.02 ± 0.34 h, respectively. However, the difference was not statistically significant because of the large variations in this measurement, it was reproducible. Half-life of IFN-λ1 mRNA in MuV-infected cells was tended to decrease by PKR-KD, but that of IFN-β mRNA was not altered. These results suggest that PKR-dependent SGs formed by MuV infection stabilize IFN-λ1 mRNA.

### SGs Partly Suppressed IFN-λ Production, but Did Not Affect Viral Replication

**Fig 6:**
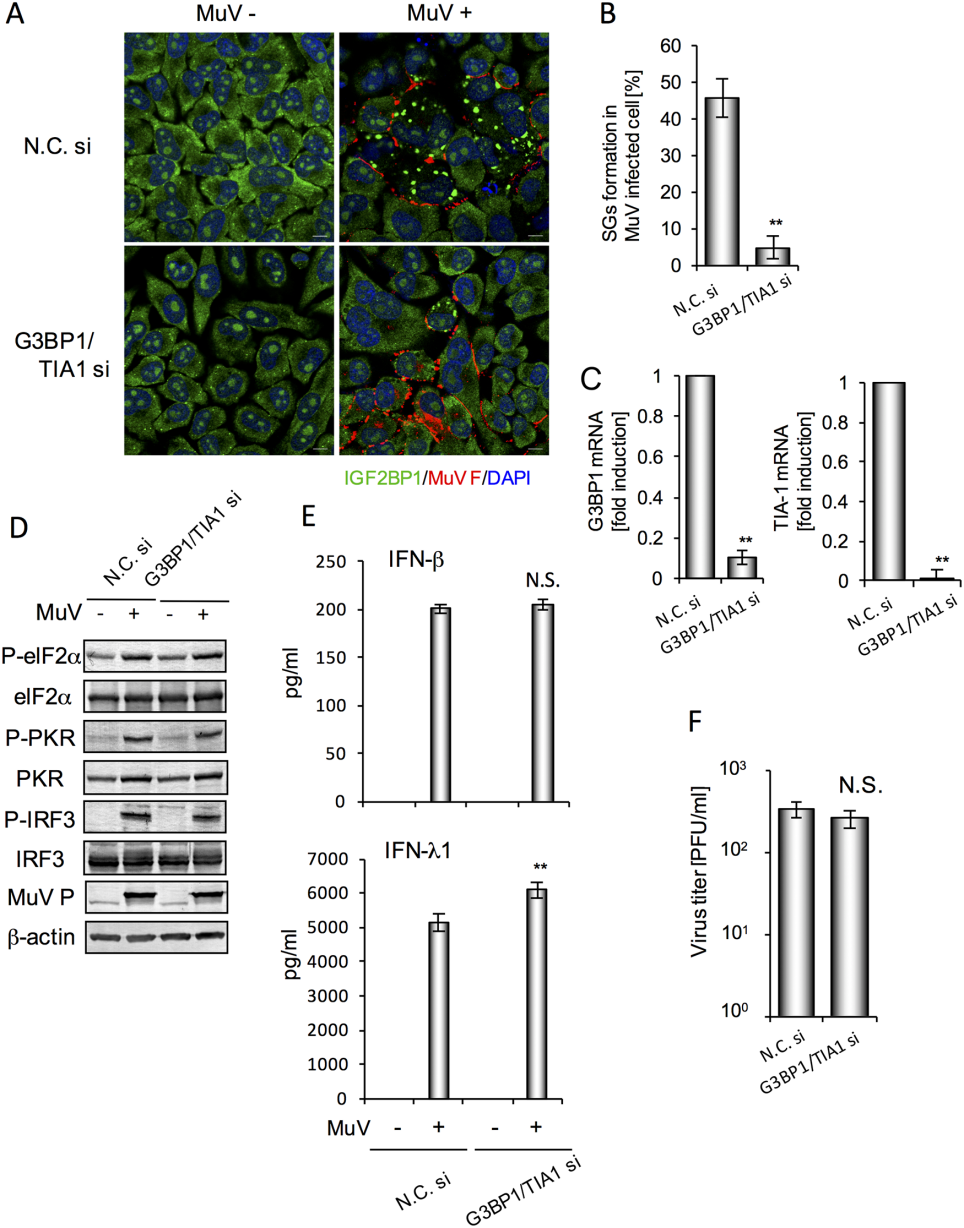
G3BP1 and TIA-1 double-KD cells, which had decreased MuV-induced SGs, partly increased MuV-induced IFN-λ1 production, but did not affect MuV replication. FL cells were transfected with targeting siRNA (G3BP1 si and TIA-1 si) or nontargeting control siRNA (N. C. si) for 48 h. (A) Immunofluorescence staining of IGF2BP1 (green) and MuV F (red) proteins and staining with DAPI (blue). Scale bar indicates 10 μm. (B) Quantification of foci containing IGF2BP1 in MuV-infected cells observed by immunofluorescence microscopy (*n* = 3). F-protein-positive cells were counted as MuV-infected cells. (C) Real-time RT-PCR analysis in G3BP1 and TIA-1 double-KD cells and control cells transfected with G3BP1 siRNA/TIA-1 siRNA and N.C. siRNA, respectively, at 48 h post-transfection (*n* = 3). (D) Phosphorylation status of PKR, eIF2α and IRF3 analyzed by western blotting. β-Actin was determined as a control. (E) Amounts of IFN-β and IFN-λ1 in culture supernatants (*n* = 4) at 24 h post-infection determined by ELISA. (F) Viral titer in supernatants (*n* = 4) at 24 h post-infection. The bar graphs represent means ± standard deviation. *P<0.05, **P<0.01, N.S.: not significant.

To investigate the contribution of MuV-induced SGs to IFN production and viral replication, we used cells with KD of the typical SG components, G3BP1 and TIA-1. SGs containing IGF2BP1 protein induced by MuV infection (24 h post-infection) were only reduced by ~50% in TIA-1- or G3BP1-KD cells compared with control cells, although mRNA expression was decreased to a greater extent (data not shown). Therefore, we prepared double TIA-1/G3BP1-KD cells, and MuV-infection induced SGs in 4.7% of these cells, compared with 45.6% of control cells (Fig 6A and 6B). We confirmed that SGs containing HuR protein were highly decreased by TIA-1 and G3BP1 double-KD (data not shown). Moreover, TIA-1 and G3BP1 mRNA levels were decreased to <10% (Fig 6C). MuV-induced phosphorylation of eIF2α and PKR was comparable in control and double-KD cells (Fig 6D). IFN-λ1, but not IFN-β, production induced by MuV infection was significantly higher in the double-KD cells than control cells (Fig 6E); however, MuV-induced phosphorylation of IRF3 and viral replication were not significantly altered by double-KD (Fig 6D and 6F). These data indicated that MuV-induced SGs suppressed IFN-λ production to some extent; however, they did not influence viral replication in these particular experimental conditions.

**Fig 7:**
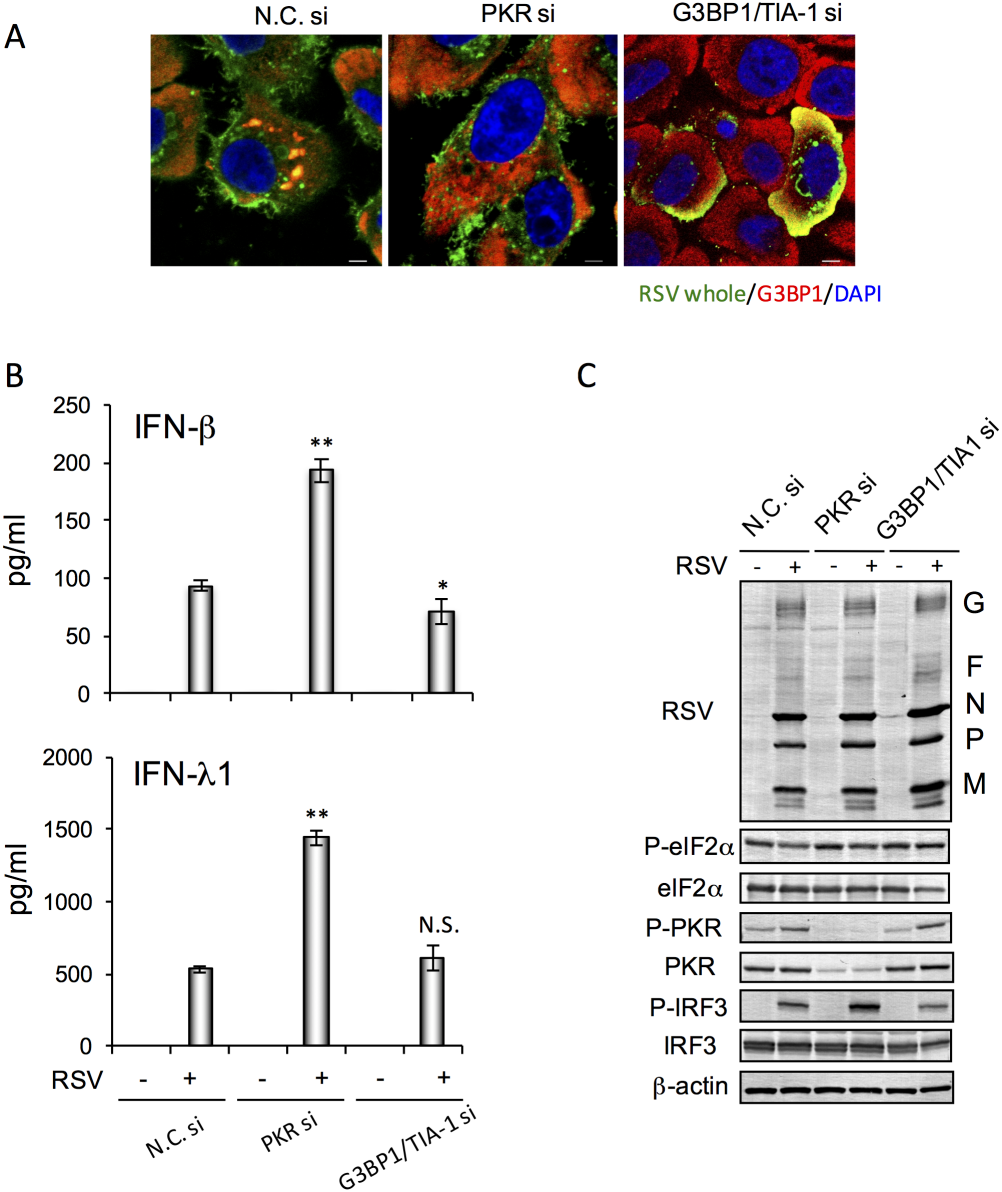
RSV-induced SGs and IFN production were higher in PKR-KD cells, but not in G3BP1/TAI-1-double-KD cells, than control cells. BEAS-2 cells were transfected with targeting siRNA (PKR si, or G3BP1 si/TAI-1 si), or nontargeting control siRNA (N.C. si) for 48 h. Cells were infected with RSV at MOI 10. (A) Immunostaining with RSV proteins (green) and G3BP1 (red), and staining with DAPI (blue) of the cells at 24 h post-infection. Scale bar indicates 5 μm. (B) Amounts of IFN-β and IFN-λ1 in the culture supernatants (*n* = 4) at 24 h post-infection, determined by ELISA. (C) Expression of RSV proteins, and phosphorylation status of PKR, eIF2α and IRF3 determined by western blotting. β-Actin was examined as a control. The bar graphs represent means ± standard deviation. *P<0.05, **P<0.01 versus RSV-infected control (N.C. siRNA transfected) cells.

We examined whether SGs were related to induction of IFNs in RSV-infected BEAS-2 cells (Fig 7). RSV-induced SGs were formed in a PKR-dependent manner (Fig 7A). RSV infection markedly induced IFN-β and -λ1 production. The amount of IFNs induced was significantly higher in PKR-KD cells than control cells, but it was not altered in G3BP1 and TIA-1 double-KD cells compared with control cells (Fig 7B). Neither PKR-KD nor double-KD cells formed SGs in response to RSV infection (Fig 7A). Expression of viral proteins was not altered in these KD cells compared with control cells (Fig 7C). These results indicated that RSV-induced SGs did not interfere with IFN production and viral replication; however, PKR suppressed RSV-induced IFN protein production.

## Discussion

We demonstrated that MuV induced SGs in a PKR-dependent manner and strongly induced IFN-β (type I) and IFN-λs (type III). MuV was recognized by RIG-I and activated IRF3 via MAVS, resulting in IFN production. IFN-λs were strongly stimulated by MuV infection, more than IFN-β.

IFN-λ1 [interleukin (IL)-29], IFN-λ2 (IL-28A) and IFN-λ3 (IL-28B) were discovered by two independent groups in 2003 [31, 32]. IFN-λs activate the JAK–STAT pathway through a receptor consisting of IFN-λ receptor 1 (IFNLR1) and IL-10 receptor 2 and induce ISGs. IFNLR1 expression is restricted to epithelial cells; thus, sensitivity of IFN-λs is limited to epithelial cells [33]. IFN-α/β can affect all types of cells, because the IFN-α/β receptor (IFNAR) consisting of IFNAR1 and IFNAR2 is ubiquitously expressed on them. Several studies have reported that antiviral potency of IFN-β and -λs is comparable [34, 35]. IFN-λs play a major antiviral role against rotavirus infection [36]. The present study showed that MuV replication increased in RIG-I- or MAVS-KD cells, which produced dramatically fewer IFNs, including IFN-λs (Fig 3). This suggests that IFN-λs restrict MuV replication, similar to IFN-α/β, in epithelial cells.

We found that protein and mRNA levels of IFN-λ1 induced by MuV were markedly higher than those of IFN-β (Fig 4). IFN-λ1 promoter contains transcription factor binding motifs of GATA binding protein 1 (GATA-1), IRF3 and NF-κB. IRFs and NF-κB are necessary for maximum expression induced by poly I:C, a mimic compound of viral dsRNA [37]. IFN-β promoter contains binding motifs of activator protein-1 (AP-1), NF-κB, and two IRFs, and IRFs and NF-κB are necessary for maximum expression, as well as IFN-λ [38]. IRFs and NF-κB were significantly stimulated by MuV infection (Fig 4B). The difference in expression levels of IFN-λ1 and -β proteins might be explained by mRNA stability. Stability of mRNA is regulated by RNA-binding proteins [39]. IL-6 mRNA is stabilized and degraded by AT-rich interactive domain-containing protein 5A and Regnase-1, respectively [40, 41]. IFN-β mRNA is stabilized by HuR protein, which binds to A/U-rich elements on 3′ untranslated regions [18, 42]. Prokunia-Olsson et al. reported that IFN-λ mRNA was still detectable 24 h after poly I:C stimulation [43]. In contrast, type I IFN mRNA expression seemed to be transient, and was decreased within 10 h after viral infection [44]. In MuV-infected cells, half-life of IFN-λ1 mRNA was 2-fold longer than that of IFN-β mRNA. The higher production of IFN-λ1 compared with IFN-β might be related to the prolonged half-life of its mRNA (Fig 5). In addition, the half-life of IFN-λ1 mRNA was shorter in PKR-KD cells. We anticipated as a working hypothesis that PKR-dependent SGs act to sequester IFN-λ1 mRNA in MuV-infected cells. Although it is conceivable that the half-life was regulated by PKR-related RNA-binding proteins, this was complicated by the fact that PKR-KD cells expressed higher levels of IFN-λ1 proteins following induction by MuV infection. However, the mRNAs could be sequestered and stabilized in PKR-dependent SGs and not be used for translation in MuV-infected cells.

There are three types of SG formation depending on virus species: stable formation, transient formation, and no formation [10]. MuV infection induces SGs, which were observed in ~85% of MuV-infected cells. Thus, MuV might induce stable formation of SGs. MeV, SeV and IAV do not form SGs, whereas mutant viruses with deleted nonstructural proteins do form SGs. Their nonstructural proteins prevent PKR activation [18–20, 45, 46]. C protein deletion mutant of MeV forms SGs, and the SGs stimulate IFN-β production and suppress viral replication [19]. NS1 deletion mutant of IAV also induces SGs and produces excess IFN-β, resulting in suppression of viral RNA replication and protein synthesis [18, 46]. These SGs interfere with IFN production and play an antiviral role. However, it is still not completely understood whether SG formation interferes antivirally or provirally. Hepatitis C virus (HCV) induces SGs in a PKR-dependent manner. HCV-induced SGs suppress translation of ISG proteins, such as MxA and ubiquitin-specific protease 18, and are required for assembly and egress of viral particles [47]. Suppression of RSV-induced SG formation by PKR-KD suggests that SGs do not interfere directly with viral replication, while IFN-β production is suppressed [48, 49]. However, suppression of RSV-induced SG formation by G3BP1-KD suggests that SGs facilitate viral replication [50]. These SGs play a proviral role. In the present study, RSV-induced PKR-dependent SGs suppressed IFN-λ1 and -β production, whereas suppression of SG formation by KD of G3BP1 and TIA-1 did not alter IFN production (Fig 7B). The relationship between SGs and IFN production/viral replication seems to be complicated.

MuV infection induced strong PKR phosphorylation and weak eIF2α phosphorylation. Although production of IFN, especially IFN-λ, was potentially suppressed by SGs induced by MuV infection in a PKR-dependent manner, the SGs did not promote viral replication. There are two possible reasons why SG-dependent suppression of IFNs had no effect on viral replication. (i) PKR induces SG formation in viral infection, and has antiviral activity, as an ISG [51, 52]. For example, PKR phosphorylates eIF2α, which prevents the generation of viral proteins by transient stalling of translation. (ii) MuV-infected cells do not respond to IFNs, because MuV V protein strongly suppresses IFN signal transduction through degradation of STAT1 [53, 54]. Nevertheless, MuV-induced PKR activation and SG formation might partly prevent IFN production; however, suppression of IFNs might be insufficient for suppression of viral replication in the experimental infection system used in this study. The role of PKR and its relationship with IFN production in MuV infection are still not fully understood; therefore, we need to elucidate this mechanism in a future study.

In conclusion, MuV induces SGs in a PKR-dependent manner and partly suppresses MuV-induced IFN production. MuV infection induces type I and III IFNs, and RIG-I and MAVS are important molecules for IFN production. Our results promote understanding of the relationship between virus-induced SGs and the innate immune response; however, further studies are needed to understand the role of SGs and innate immune responses against viral infection, including mumps.
